# Overexpression of TIMP-3 in Chondrocytes Produces Transient Reduction in Growth Plate Length but Permanently Reduces Adult Bone Quality and Quantity

**DOI:** 10.1371/journal.pone.0167971

**Published:** 2016-12-21

**Authors:** Blandine Poulet, Ke Liu, Darren Plumb, Phoung Vo, Mittal Shah, Katherine Staines, Alexandra Sampson, Hiroyuki Nakamura, Hideaki Nagase, Alessandra Carriero, Sandra Shefelbine, Andrew A. Pitsillides, George Bou-Gharios

**Affiliations:** 1 Department of Musculoskeletal Biology, Institute of Ageing and Chronic Disease, University of Liverpool, Apex building, Liverpool, United Kingdom; 2 Kennedy Institute of Rheumatology, Oxford, United Kingdom; 3 Comparative Biomedical Sciences, The Royal Veterinary College, London, United Kingdom; 4 Department of Biomedical Engineering, Florida Institute of Technology, Melbourne, FL, United States of America; 5 College of Engineering, Northeastern University, Boston, MA, United States of America; University of Texas Southwestern Medical Center, UNITED STATES

## Abstract

Bone development and length relies on the growth plate formation, which is dependent on degradative enzymes such as MMPs. Indeed, deletion of specific members of this enzyme family in mice results in important joint and bone abnormalities, suggesting a role in skeletal development. As such, the control of MMP activity is vital in the complex process of bone formation and growth. We generated a transgenic mouse line to overexpress TIMP3 in mouse chondrocytes using the Col2a1-chondrocyte promoter. This overexpression in cartilage resulted in a transient shortening of growth plate in homozygote mice but bone length was restored at eight weeks of age. However, tibial bone structure and mechanical properties remained compromised. Despite no transgene expression in adult osteoblasts from transgenic mice *in vitro*, their differentiation capacity was decreased. Neonates, however, did show transgene expression in a subset of bone cells. Our data demonstrate for the first time that transgene function persists in the chondro-osseous lineage continuum and exert influence upon bone quantity and quality.

## Introduction

Bone formation is a complex three-dimensional process in which several signaling molecules play a role in controlling skeletal development. Crucially, components of the extracellular matrix (ECM) and their remodelling by matrix metalloproteinases (MMP) play critical roles in cartilage and bone structure and function. The process of endochondral ossification, which is essential for bone formation and growth, involves a cartilage intermediate. During this process, mesenchymal condensation differentiates into a cartilage template, rich in collagen type II. Chondrocytes become hypertrophic and produce a calcified cartilage matrix, which is finally replaced by bone matrix, rich in collagen type I, produced by invading osteoblasts.

This remodelling of cartilage is dependent on degradative enzymes such as MMP. Deletion of these enzymes in mice result in important joint and bone abnormalities, suggesting a role in skeletal development [1–3]. As such, the control of their activity is vital in the complex process of bone formation and growth. Although deletion of some regulators of matrix remodeling do not seem to affect bone development severely, they nonetheless result in pathologies that remain undisclosed. One such example is the deletion of Tissue inhibitor of metalloproteinases (TIMP), which are the major natural inhibitors of MMPs. In particular TIMP3 has been shown to be able to inhibit the activity of MMPs and A Disintegrin and Metalloproteinase (ADAMs) [4, 5]. In addition, TIMP3 was shown to be expressed in embryonic and early postnatal stages in tissues undergoing extensive remodelling and in adult tissues with rapid turnover [6–12]. This therefore suggests a role of TIMP3 in controlling matrix turnover in various tissues. Although TIMP3 deletion did not appear to induce any overt skeletal pathology [13], no in depth analysis of the early bone development and adult bone phenotype have yet been described.

The mechanical properties of bones are essential to maintain their function to withstand loads and changes in the bone’s structure and mass can alter these properties. Thus bone development and growth, which involves a cartilage intermediary through endochondral ossification or growth plate, has a direct impact on the strength of bones in later life. Our aim is therefore to use murine transgenic approaches to define the importance of TIMP3 expressed by chondrocytes on bone development and function in adults. Mice that overexpress TIMP3 specifically in chondrocytes under the control of the promoter/enhancer collagen type II (Col2a1) gene were used and we find major defects in bone formation and in bone structure and mechanical properties in adult mice, which suggests a control of bone by chondrocytes, possibly via the transdifferentiation of growth plate hypertrophic chondrocytes into bone cells.

## Results

### High levels of TIMP3 overexpression are lethal postnatally.

**Fig 1:**
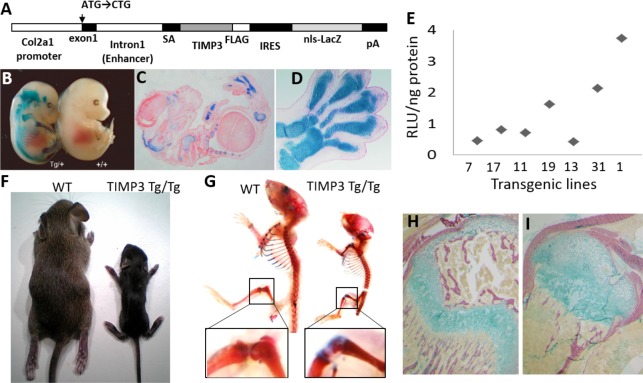
High TIMP3 overexpression in mice from line 1 induced defective skeletal development and growth. A, Diagram depicting the transgene utilized for overexpression of TIMP3 restricted to chondrocytes using the Col2a1 promoter and enhancer, followed by LacZ. B-D, LacZ staining showing expression of the transgene in TIMP3 Tg/+ in the cartilaginous skeletal elements in whole-mount embryos (B) and histological sections across the whole embryo (C; magnification x4) and in the developing forelimb (D; magnification x10); E, TIMP3 protein amount (relative light unit (RLU) per ng of total protein) in each transgenic line created. F-I, severe skeletal phenotype in TIMP3 Tg/Tg line 1 with high expression of the transgene, with significant differences in overall pup size at P14 (F), in whole mount pups stained with Alizarin red and Alcian blue and magnified view of the knee (G) with delayed ossification of the epiphysis with increased amount of cartilage tissue (blue). Histological sections of knee joint epiphysis stained with Alizarin red and Alcian blue in WT (H) and TIMP3 Tg/Tg (I) showed lack of secondary ossification centre in the transgenic mouse (Magnification x10).

Transgenic mice exploiting a 3kb promoter and 3kb first intron of the Col2a1 gene to drive overexpression of human TIMP3 (TIMP3 (Tg/Tg)), followed by an IRES and ß-galactosidase gene (Fig 1A) showed chondrocyte-specific expression during development (Fig 1B–1D) confirming restriction of the transgene to cartilage. Several murine lines expressing different levels of human TIMP3 were produced (Fig 1E). Those possessing highest TIMP3 overexpression levels (line 1) died within 2 weeks of birth and exhibited marked stunting of growth. Skeletal comparison of Tg/Tg mice to WT littermates revealed that most ribs and knee joints showed accumulation of matrix, which stained positively with Alcian blue for proteoglycans, as well as deficient ossification levels consistent with the failure to remodel the matrix in order to facilitate bone formation (Fig 1G). High expression of the transgene in P14 mice (14 days old postnatal) also showed restricted endochondral ossification, disorganised cartilage with excessive matrix and fewer chondrocytes in epiphyseal locations where secondary ossification centres had otherwise formed in WT mice (Fig 1I and 1H); Tg/Tg mice also showed disorganised and deficient metaphyseal trabecular bone compared with WT littermates.

### Ectopic TIMP3 transgenic expression is seen in proliferating chondrocytes of altered growth plates of TIMP3 transgenic mice

**Fig 2:**
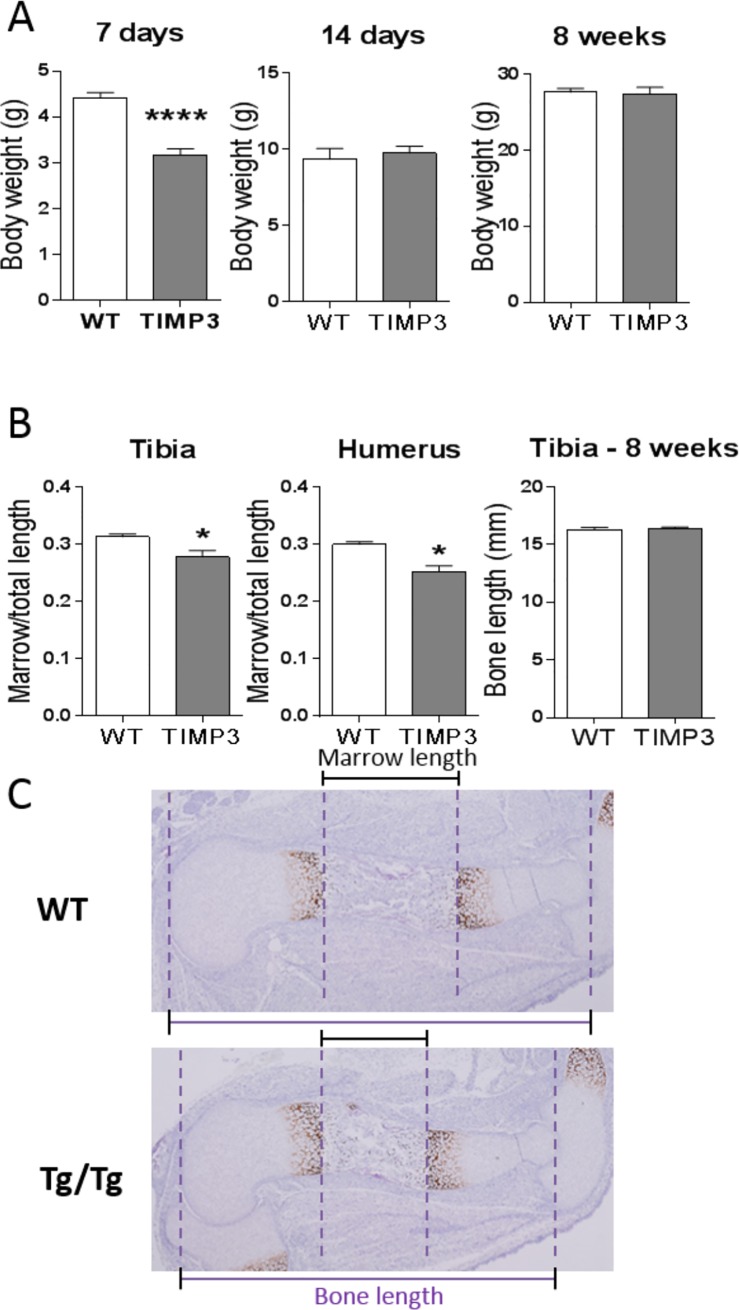
Bone and growth plate lengths are reduced with TIMP3 overexpression in young growing mice. TIMP3 Transgenic line 19 with lower transgene expression levels were used for bone analysis. A. Body weight of WT and TIMP3 Tg/Tg mice at 7 and 14 days, and at 8wks. B. Tibia and Humerus length of the marrow cavity relative to the total length at E16.5 and bone length at 8 weeks of age (n = 5). C. Histological pictures of the tibia of WT and TIMP3 Tg/Tg at E16.5, stained with Collagen type 10 to show the edges of the marrow cavity. Dashed lines show the edges of the whole bone (outer lines) and edges of the marrow cavity (middle lines). Student’s t-test was used for statistical analysis (* for p<0.05, **** for p<0.0001).

Amongst the different TIMP3 transgenic mouse, another line (line 19), expressed an intermediate level of the transgene and survived into adulthood. It was found that breeding of this line to homozygosity disclosed early defects in bone length and growth plate organisation. Indeed, TIMP3 Tg/Tg mice (line 19) were identifiable by virtue of being born smaller up to P7 (Fig 2A), with shorter limbs compared to WT controls (Fig 2B and 2C). This difference in size, however, was no longer visible at P14 and 8 weeks of age.

**Fig 3:**
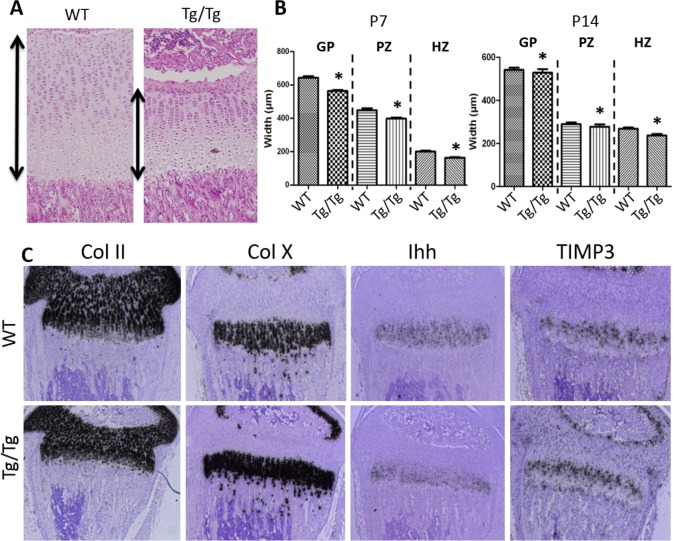
Thinner growth plates support bone length shortening in growing TIMP3 Tg/Tg mice (Line 19). A, histological representation stained with H&E of the growth plate from WT and TIMP3 Tg/Tg mice at P14 (Magnification x10). B, quantification of the width of the growth plate (GP), the proliferating zone (PZ) and hypertrophic zone (HZ) at P7 and P14. (n = 5; Student’s t-test; * for p<0.05). C, In situ hybrization for Collagen type II (Col II, in all chondrocytes), Collagen type X (Col X; marker of hypertrophic chondrocytes), Indian Hedghog (Ihh, found in the hypertrophic zone cells) and TIMP3 mRNA in the tibia of P14 WT (top panels) and TIMP3 Tg/Tg (bottom panels). Of importance, TIMP3 mRNA was found mainly in the prehypertophic zone cells in the WT, but we additionally found in all other chondrocytes including in the proliferating and hypertrophic zones (Magnification x4).

Histological analysis of their growth plates (Line 19) revealed a ~40% reduction in the width of the growth plate (Fig 3A) with marked decreases in the proliferating and hypertrophic zones (Fig 3B). In contrast, heterozygous TIMP3 transgenic mice were not affected (data not shown). *In situ* hybridisation (Fig 3C) for collagen type II and X mRNA in P14 mouse tibiae showed a deficit in the width of both proliferative and hypertrophic growth plate zones in Timp3 Tg/Tg mice, respectively. Indian hedgehog and parathyroid related protein mRNA (not shown) were distributed similarly with likewise intensity in both Timp3 Tg/Tg and WT mice, albeit in a reduced area of the hypertophic zone. TIMP-3 mRNA was restricted to the pre-hypertrophic zone in WT mice but was additionally expressed in the proliferative zone in the Tg/Tg mice (Fig 3C). Ectopic expression of neither collagen type II nor TIMP-3 transgene was seen in the subchondral bone or along the cortical bone length (data not shown). Line 19 mice were used for the rest of this study.

### Col2a1-driven TIMP3 overexpression decreases adult murine bone mass.

**Fig 4:**
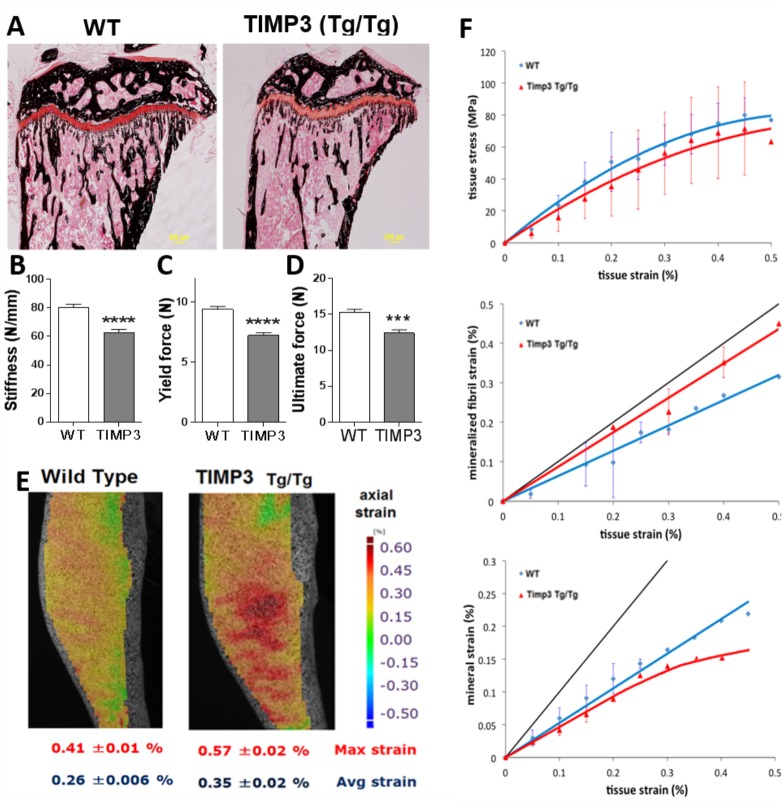
TIMP3 overexpression decreases tibial bone mechanical properties from macroscale to nanoscale mechanics. A, Von Kossa staining of tibia epiphysis and metaphysis of WT and TIMP3 Tg/Tg mice showed decreased trabecular bone in TIMP3 Tg/Tg mice (Magnification x2). B-D Mechanical properties of the femur showing decreased Stiffness, yield force and ultimate force in TIMP3 Tg/Tg mice (Student’s t-test; *** for p<0.001, **** for p<0.0001; n = 8 WT, n = 12 Tg/Tg). E, Axial strain heat map under compression along the tibial shaft (n = 4). F, Strain curves against tissue stress, mineral fibril strain and mineral strain in WT (blue) and TIMP3 Tg/Tg (red; n = 5).

Tibiae from 8 week-old mice were micro-CT scanned and analysed. This showed that bone in the trabecular compartment of TIMP3 Tg/Tg mice exhibited significantly lower mass, with decreased BV/TV and trabecular thickness, and modification in bone structure with elevated trabecular pattern factor and decreased trabecular number (Table 1). Von Kossa staining confirmed decreased trabecular bone mass in TIMP3 Tg/Tg mice (Fig 4A). Similarly, analysis of cortical bone by microCT showed decreased total bone area, bone perimeter, MMI (Mean polar moment of inertia) and medullary area (Table 1). These data indicate that TIMP3 overexpression driven via Col2a1 modifies trabecular and cortical bone mass and architecture.

**Table 1:** Trabecular and cortical structure is modified in TIMP3 Tg/Tg and WT mice at 8wks of age.

Trabecular	**WT**	**TIMP3 Tg/Tg**	**p-value**
**BV/TV**	**12.12 (±0.66)**	**8.49 (±0.57)**	***0*.*0014***
**Tb.Th**	**0.052 (±0.001)**	**0.046 (±0.001)**	***0*.*0036***
**Tb.Sp**	0.24 (±0.015)	0.26 (±0.018)	*0*.*3801*
**Tb.N**	**2.32 (±0.12)**	**1.81 (±0.09)**	***0*.*0106***
**Tb.Pf**	**27.66 (±0.69)**	**33.69 (±1.14)**	***0*.*0009***
**SMI**	2.20 (±0.06)	2.38 (±0.02)	*0*.*0685*
Cortical	**WT**	**TIMP3 Tg/Tg**	**p-value**
**T.Ar**	**1.11 (±0.02)**	**0.90 (±0.03)**	***<0*.*0001***
**T.Pm**	**4.11 (±0.04)**	**3.69 (±0.06)**	***<0*.*0001***
**B.Ar**	**0.73 (±0.01)**	**0.61 (±0.02)**	***<0*.*0001***
**MMI(polar)**	**0.18 (±0.007)**	**0.12 (±0.007)**	***<0*.*0001***
**Ecc**	0.55 (±0.01)	0.56 (±0.01)	*0*.*6326*
**Cs.Th**	0.22 (±0.003)	0.21 (±0.007)	*0*.*0716*
**M.Ar**	**0.37 (±0.01)**	**0.29 (±0.02)**	***0*.*0023***

BV/TV: Bone Volume/Total Volume (%); Tb.: Trabecular; Th.: Thickness (mm); Sp.: Separation(mm); N.: Number (1/mm); Pf.: Pattern Factor (1/mm); SMI: Structure Model Index; TA: Total Area (mm2); T.Pm: Total Perimeter (mm); BA: Bone area (mm2); MMI(polar): Mean polar moment of inertia (mm4); Ecc: Mean eccentricity; Cs.Th: Cross-sectional thickness (mm); MA: Medullary Area (mm2). Statistical significance measured by Student’s t-test. N = 9 TIMP3 Tg/Tg, n = 17 WT.

### Bone mechanical properties are compromised in Col2a1-driven TIMP3 Tg/Tg mice.

To examine whether this diminution of mass and compromised bone structure in Col2a1-driven TIMP3 Tg/Tg mice also influenced bone’s functional load-bearing qualities, 3-point bending tests were performed on femurs of 8wk-old mice and showed that stiffness, yield load and ultimate load were all significantly lower in TIMP3 Tg/Tg compared to WT control mice (Fig 4B–4D).

In addition, the spatial strain distribution over the medial side of the tibia was calculated, based on the three components of displacement measured using a digital image correlation system. Fig 4E shows only the strain in the axial (loading) direction, as transverse strain and shear strain both had relatively low magnitude in comparison. Strain maps indicate a non-uniform strain field across the surface of the tibia, with the axial compression resulting in tension on the medial tibia because of its curved shape, in agreement with previous studies [14]. Consistent with compromised load-bearing strength in bones of these TIMP3 Tg/Tg mice, load-engendered tissue strains were increased in magnitude and showed modification in their pattern compared with WT mice. Specifically, the medial surface of the tibia of TIMP3 Tg/Tg mice had a maximum strain of 0.57 ± 0.02% vs. 0.41 ± 0.01% in WT, with an average strain of 0.35 ± 0.02% vs 0.026 ± 0.006%, respectively. Load-deformation curves were similar both during each repeated loading episode and for all mice within each genotype, indicating there was no load-related failure. However, TIMP3 Tg/Tg had a 20% greater compressive extension than WT.

In order to resolve the contribution of mineralized collagen fibrils and mineral crystals to overall tissue strain in bone of TIMP3 Tg/Tg mice, we coupled *in situ* tensile testing of bone with simultaneous small angle X-ray scattering (SAXS) and wide angle X-ray diffraction (WAXD). These data showed that at small tissue strains, bone from TIMP3 Tg/Tg mice has increased fibril strain, implying that the fibrils are less stiff compared with WT (Fig 4E). Differences in fibril nanomechanics are possibly caused by lower levels of inter-fibrillar shear transfer due to reduced mineralization.

As these bone qualities are likely a functional property of osteoblasts or osteocytes, not chondrocytes, these data together provide strong indication that the TIMP3 transgene driven by chondrocyte-selective Col2a1 exerts either direct or indirect effects on bone mass and quality. Alternatively, these data may indicate that the influence of a Col2a1-driven transgene persists across the newly appreciated chondro-osseous lineage continuum to functional impact osteoblast behavior.

### Osteoblasts from *Col2a1*-driven TIMP3 Tg/Tg mice exhibit modified behaviour *in vitro*.

**Fig 5:**
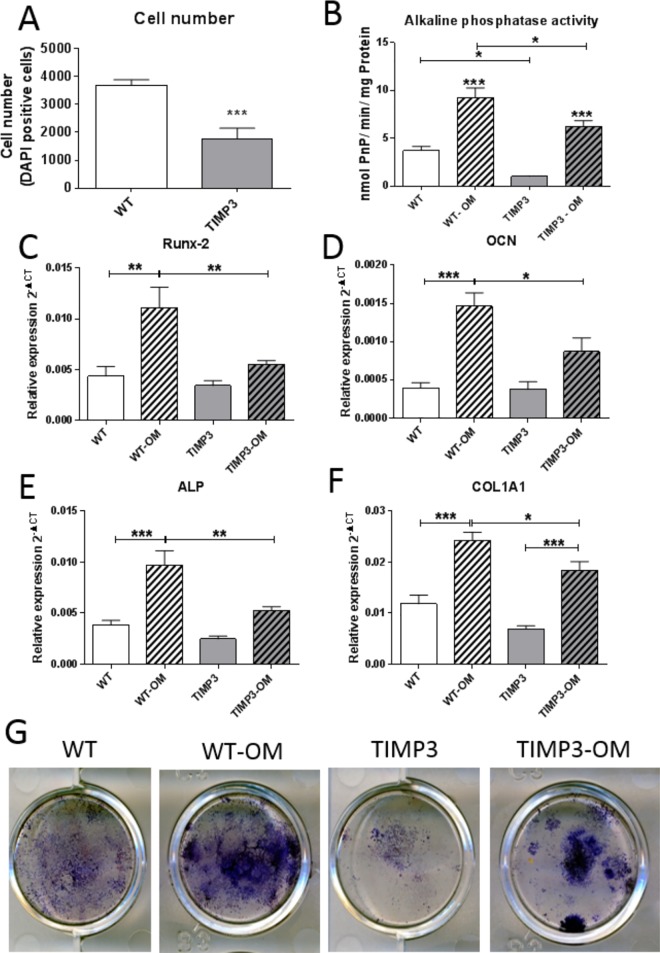
Primary osteoblasts culture from WT and TIMP3 Tg/Tg mouse tibiae at 8wks of age showed decreased cell number and differentiation capacity. A. Cell number was decreased in TIMP3 Tg/Tg mice. B-F, alkaline phosphatase activity (B) and mRNA expression of RUNX-2 (C), Osteocalcin (OCN, D), Alkaline phosphatase (ALP, E) and Collagen type I (COL1A1, F) in WT (white bars) and TIMP3 Tg/Tg (grey bars) mouse osteoblasts with and without osteogenic medium treatment (OM, to induce osteogenic differentiation, hashed bars), showing an increase in expression in all genes with osteogenic differentiation in WT cell, compared to a significant stunted promotion of these genes during differentiation in TIMP3 Tg/Tg cells. G, representative images of Alkaline phosphatase activity visualised using Fast Blue dye. (3 replicate wells from 3 plates per treatment and per genotype taken from a total of n = 4 WT and n = 6 Tg/Tg mice; One Way ANOVA test; * for p<0.05; ** for p<0.01; *** for p<0.001).

To define whether *Col2a1*-driven TIMP3 overexpression modified osteoblast function, behaviour of primary osteoblasts extracted from tibiae of 8wk-old WT and TIMP3 Tg/Tg mice were compared. Consistent with lower rates of growth, osteoblasts from TIMP-3 Tg/Tg mice were found in significantly lower numbers than cells from WT mice after an extended culture in vitro. (Fig 5). Furthermore, alkaline phosphatase activity and mineralisation were also both significantly lower in osteoblasts from TIMP3 Tg/Tg bone. Under osteogenic conditions, markers of osteoblastic differentiation, including Runx-2, alkaline phosphatase, osteocalcin and collagen type I mRNA expression levels were all significantly lower (Fig 5) in osteoblasts from TIMP3 Tg/Tg compared with WT osteoblasts; levels of none of these mRNA transcripts differed in WT and TIMP3 Tg/Tg osteoblasts in non-osteogenic control conditions. These data suggest that osteoblasts isolated from TIMP3 Tg/Tg mice, maintained in isolation of *Col2a1*-driven transgene expressing chondrocytes, have perturbed osteogenic potential. Cartilage markers, Sox9 and aggrecan, as well as expression of the transgene detected by LacZ reporter were not detectable in any of these osteoblast cultures.

**Fig 6:**
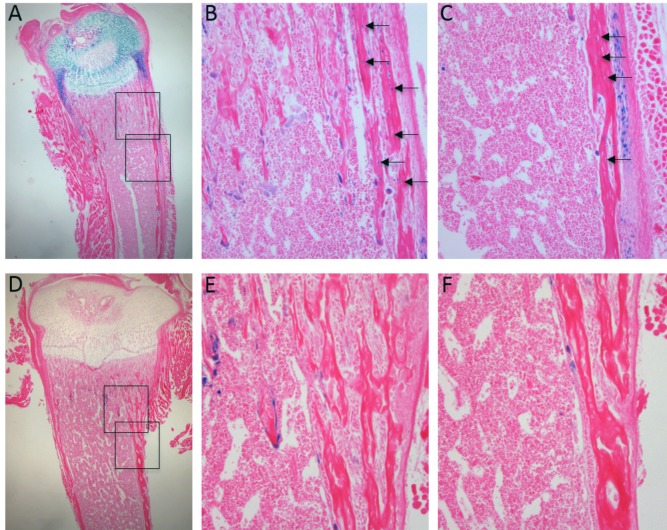
Beta-galactosidase staining from the TIMP3-LacZ transgene expression on the Col2a1 promoter/enhancer was found in bone cells at P11. Cells expressing the trangene-LacZ stained blue (beta-galactosidase) and were found in the epiphyseal cartilage (A) and in the trabecular (B) and cortical (C) bone cells (arrows). D-F show non-transgenic controls, with positive blue staining showing osteoclasts, and therefore not linked to the transgene, but no osteocytes embedded in the bone matrix were beta-galactosidase-positive. (A and D Magnification x2; B-C, E-F Magnification x20)

Finally, we interrogated the origin of this impairment in osteoblast function by exploring the distribution of the ß-galactosidase gene (LacZ) reporter in transgenic mice *in vivo*. Despite transgene overexpression via a Col2a1 promoter, cells expressing ß-galactosidase were found not only throughout cartilage, but also in some osteoblasts in both the trabecular and cortical bone compartments, and some osteocytes at P11 (Fig 6).

## Discussion

In this study we overexpressed TIMP3 using a Col2a1 promoter to achieve specificity to chondrocytes, and obtained a set of transient defects in growth plate and permanent defects in bone. Specifically, Line 19 is a mouse line that overexpressed TIMP3 transgene at low level to allow the mouse to survive during development, yet high enough to produce a transient cartilage phenotype only in homozygote mice. We found that the transgene-induced transient phenotype in the growth plate in this line 19, with shortening of the proliferating zone and overall bone length in development and early life, was followed by an effective ‘catch-up’ by eight weeks of age. In contrast, early deficiencies in structure and mechanical bone properties in the skeleton of Col2a1-driven TIMP3 transgenic mice persisted into adulthood. Although we have not defined a mechanism in this study, there are many possibilities explaining how the matrix protein TIMP3 overexpressed by chondrocytes affects osteoblast function, which are discussed below.

In agreement with previous work [6], TIMP3 mRNA levels detectable by *in situ* hybridization in WT mice were relatively low and restricted to chondrocytes in the pre-hypertrophic growth plate zone. However, TIMP3 mRNA expression was more widespread in the Col2a1-driven TIMP3 Tg/Tg, extending additionally into the proliferating growth plate zone. These changes were not linked with modified expression of the essential cartilage signaling molecules, Indian hedgehog (Ihh) or parathyroid hormone/ parathyroid hormone-related peptide (PTH/PTHrP). These data are in accord with similar findings in MMP-14 null mice [1]. In addition, we found that like MMP-14 and MMP-16 null mice, proliferating zone shortening and decreased chondrocyte proliferation likely explain the diminution in bone length [1, 2]. This contrasts, however, with the phenotype of both MMP-13 and MMP-9 null mice, in which such severe impairment in endochondral bone growth was instead characterized by marked elongation of the hypertrophic zone, along with diminished ECM remodeling, prolonged chondrocyte survival, delayed vascular recruitment and similarly defective trabecular bone formation [3]. It is therefore possible that the skeletal development phenotype we observed in TIMP3 transgenic pups was the product of relatively elevated levels of local inhibition of MMP activity. This would be consistent with proposed roles for MMPs and TIMPs in skeletal development with both the function of TIMP3 as a matrix-bound inhibitor of both aggrecanases such as ADAMTS (A Disintegrin And Metalloproteinase with ThromboSpondin motifs) and MMP molecules [5] and as a pivotal controller of normal cartilage matrix turnover [4, 15].

As well as playing an important role in skeletal development, MMP and TIMPs affect bone growth and remodeling, contribute to modulation of bone mass in response to mechanical loading and are involved in fracture healing and in osteoblast-to-osteocyte transition [16–22]. TIMP3 overexpression in chondrocytes and its matrix-enrichment may therefore exert paracrine actions on MMP activity in bone that could explain the decreased bone mass and thinner cortices found in this Col2a1-driven TIMP3 Tg/Tg. Indeed, decreased bone mass has also been observed in mice deficient in certain MMPs, including MMP-9 and MMP-14 as well as in MMP-9/-13 double knock out mice [1, 23]. In addition, MMP-13 null mice also exhibit decreased bone quality and increased fracture risk [3, 18]. Our data suggest that the bone phenotype seen in Col2a1-driven TIMP3 Tg/Tg mice is a product of increased paracrine MMP inhibition in bone by TIMP3 and that ECM TIMP3 is an important regulator of bone remodeling.

Only few studies have reported an adult bone phenotype in chondrocyte-specific murine mutants. Conditional deletion of MMP-13 in chondrocytes failed to reproduce the shorter bone and increased bone volume observed in global MMP-13 null mice, suggesting that chondrocyte-specific loss of MMP-13 is not sufficient to explain this modification in bone architecture [3, 24]. Unsurprisingly, chondrocyte-selective deletion of collagen type II resulted in smaller mice with shorter bones and thinner trabeculae [25]. Interestingly, a recent paper describes the role of chondrocyte-specific ATF4 (Activating Transcription Factor 4) in bone development and architecture [26]. Akin to our findings, the authors showed that chondrocyte-selective ATF4 deficiency (as opposed to TIMP3 overexpression) also delayed formation of ossification centers, resulted in short bones and also decreased bone mass and osteoblast differentiation; which were all rescued by cartilage-selective ATF4 overexpression. Although the mechanisms of action were found to involve Ihh in these chondrocyte-selective ATF4 gain-of/ loss-of-function mice and, therefore, likely differ from those in our TIMP3 Tg/Tg mice, these data suggest that transgene overexpression using a cartilage-targeted promoter can indeed affect bone structure and function. Our study extends these observations suggesting that these hitherto ‘off-target’ effects impact bone structure and function in adults.

Crosstalk between cartilage and bone has been described previously. Indeed, co-culture of chondrocytes with osteoblasts has been found to restrict mineralization [27]. In addition, it has been suggested that such crosstalk via the transfer of proteins can also occur between articular cartilage and subchondral bone in osteoarthritic joints *in vivo* [28]. It has also been shown that Ihh and other factors secreted by cartilage can affect osteoblast differentiation and that fibroblast growth factors, BMPs and Wnts might also contribute similarly to this crosstalk [26, 29]. This supports the possibility that TIMP3 expressed by chondrocytes may act on osteoblasts and bone via a paracrine route. Insulin growth factor-1 (IGF-1) is one such signaling molecule that is expressed by chondrocytes that may affect bone cells. Indeed, chondrocyte-targeted deletion of IGF-1 (using the identical Col2a1 promoter used herein) produced a bone phenotype resembling that which we observed in TIMP3-Tg/Tg mice, with reduced bone length, periosteal and endosteal perimeters [30]. However, further studies are needed to determine whether the bone phenotype described herein is a result of the embryonic defects due to TIMP-3 overexpression or a direct effect of chondrocytes on bone cells and tissue.

Finally and most intriguingly, our studies unveil an impaired osteogenic potential in osteoblasts isolated from Col2a1-driven TIMP3 Tg/Tg mouse bone, maintained *in vitro*. Isolation from both the matrix and any paracrine control from the Col2a1-targeted chondrocytes points, however, to an alternative explanation for the adult bone phenotype in the Tg/Tg mice. Any alternative explanation would need also to accommodate the lack of any detectible collagen type II mRNA or TIMP3 transgene expression in these isolated osteoblasts *in vitro*. In addition, TIMP3 overexpression in bone marrow cells lead to increased bone formation, suggesting the bone phenotype described in our manuscript is not due to ectopic TIMP3 expression from hematopoietic stem cells in our system [31].

An exciting explanation appears to have been recently afforded by exciting lineage tracing experiments which have demonstrated that Col2-cre-targeted cells contributed not only to the chondrocyte population of skeletal cells but also perichondrial cells and, indeed, Col1-GFP positive osteoblasts, osteocytes and Cxcl12 high stromal cells in marrow in mice at postnatal day 3 [32]. Pulse chase studies also showed that histologically-tracked Col2-cre-ER, as well as Aggrecan-cre-ER (system including tamoxifen-dependent Cre recombinases) labelled cells contribute to osteoblast and stromal cell populations. In our study, staining in Col2a1-driven TIMP3 Tg/Tg mouse bones for ß-galactosidase, an integral component of the TIMP3 transgene ‘cassette’ expressed only whilst Col2a1 is actively transcribed, did not disclose any positive labelling of osteoblasts in 8 week-old mice. In contrast, many bone cells in both trabecular and cortical bone compartments were indeed ß-galactosidase positive in P11 newly born Col2a1-driven TIMP3 Tg/Tg mice and this was never associated with detectible TIMP3 or Col2 mRNA. Together with recent tracking studies, our data suggest that transient TIMP3 overexpression, at the early Col2a1-expressing phase in this chondrocyte-to-osteoblast lineage continuum, engenders modification of cell behavior that persists into late osteoblast phases when neither Col2a1 nor the TIMP3 transgene is actively transcribed. Similar persistence could be reflected in the contribution of bone marrow cells, which transiently express Col2a1, to the osteoblast lineage [33] or in the chondrocyte-to-osteoblast transdifferentiation observed during bone fracture healing in adult mice [34]. Although prior studies showing modified *in vitro* osteoblast differentiation also in chondrocyte-selective ATF4-deficient mice [26], the cellular mechanism by which TIMP3 exerts its persistent impact in osteoblasts remains to be defined.

In conclusion, our data have established modification in skeletal development and maintenance in mice harbouring a Col2a1-driven TIMP3 transgene. Our observations indicate that chondrocyte-selective overexpression of TIMP3 disrupts endochondral growth, particularly during early development, and produces more persistent changes in skeletal structure which compromise the functional integrity of bone in post-natal and adult life. Our data also point to a persistence downstream impact of transient TIMP3 transgene expression which extends to influence osteoblast function within the chondrocyte-to-osteoblast lineage continuum to achieve these long-term changes in bone quantity and quality.

## Materials and Methods

### Generation of TIMP3 transgenic mice

Creation of the transgenic mice and all studies were conducted under with U.K. Home Office project licenses. A transgenic construct containing collagen IIα1 chain (*Col-2a1*) proximal promoter region (3000 bp), the first exon (237 bp), the first intron (3020 bp) [35], was used to drive expression of human *TIMP-3*, an *IRES* (internal ribosomal entry site) sequence and *LacZ* with nuclear localizing signal. Not I was used to remove back-bone vector (pBluescript) and to produce 11.3-kb fragment that was microinjected into fertilized -C57BL/10 × CBA F1 eggs. Founder mice were identified by analysis of genomic DNA with Southern blot. TIMP-3 mRNA expression in E15.5 embryo were confirmed by qRT-PCR with human TIMP-3 specific primer and probe (QuantiProbe® kit, Qiagen). Homozygous and heterozygous transgenic animals were identified with TaqMan probe for β-galactosidase (sence: 5’- GTG CAC GGC AGA TAC ACT TG-3’, antisence: 5’- AAC GGT AAT CGC CAT TTG ACC AC-3’, TaqMan probe; 5’ FAM-TCA GCC GGA AAA CCT ACC GGA TTG A–BHQ 3’) and mouse 18S (sence: 5’- GAC CAT AAA CGA TGC CGA CTG -3’, antisence: 5’- CCC TTC CGT CAA TTC CTT TAA G -3’, TaqMan probe; 5’ HEX- CTT CCG GGA AAC CAA AGT CT–BHQ 3’) as described previously [36].

Mice were housed in groups of up to 6 in individually vented cages maintained at 21 ± 2°C on a 12-hour light/dark cycle with ad libitum food (RM3; Special Dietary Systems) and water. All experimental protocols were performed in compliance with the UK Animals (Scientific Procedures) Act 1986 regulations for the handling and use of laboratory animals. Mice were monitored daily for any health and welfare issues from birth, including any possible defects or significant change in size during the first two weeks. Mice were euthanised by an approved Schedule 1 method (by rising concentration of CO2 or by cervical dislocation for adult mice).

### Micro-computed tomography (microCT) analysis

Tibiae from 8week-old male WT (n = 17) and TIMP3-Tg/Tg (n = 9) mice were fixed, stored in 70% ethanol and scanned using a microCT system (Skyscan 1176, Belgium). High resolution scans with an isotropic 5μm voxel size were attained, reconstructed using NRecon v1.6.4.0 and analysed using Dataviewer v1.4.4 and CTAn v1.11.6.0+ (Skyscan, Belgium). Cortical analysis was performed on a 0.5mm segment starting at 37% of the tibial length, and the trabecular region included 200 slices (1mm) immediately distal to the growth plate. Histomorphometric analysis was performed by Skyscan software (CT-Analyzer version 1.5.1.3; [37]). For trabecular bone analysis, regions of interest were drawn to exclude the cortical shell, and three-dimensional algorithms were used to determine relevant parameters, including: bone volume (BV) percentage, BV/total volume (BV/TV), trabecular thickness (Tb.Th), number (Tb.N) and separation (Tb.Sp), Trabecular pattern factor (Tb.Pf) and Structure Module Index (SMI). For cortical bone analysis, two-dimensional computation was used, and parameters included total area (TA), cortical area (CA), medullary area (MA), Total Perimeter (T.Pm), Cross-sectional thickness (Cs.Th), Mean polar moment of inertia (MMI), Mean eccentricity (Ecc).

### Femur three-point bending

After microCT scanning, right femurs of n = 8 WT and n = 12 TIMP3Tg/Tg mice were tested until fracture by three-point bending using a standard materials testing machine (5866 Instron, Instron, Norwood, MA, USA). Femurs were loaded at the mid-diaphysis in the anterior-posterior direction with a deflection rate of 50μm/s. Force-deflection curves were analysed with a custom program (Matlab, MathWorks Inc, MA, USA) to measure the bending stiffness (S: slope of the linear elastic deformation), the yield force (Fyield, limit between the elastic and plastic deformation) and ultimate force (Fult, maximum force sustained). The plastic (post-yield) behaviour was assessed by the ratio of plastic/total work to fracture (Rp/tW, i.e. ratio of the area under the curve from the yield point to the fracture point over the total area under the curve).

### Bone surface strains using digital image correlation (DIC)

Left and right tibiae, n = 4 mice each group, were exposed. Bones were subsequently covered with a thin layer of matt, water-based, white paint, and speckled with matt, acrylic, black ink using a high precision air brush. Legs were loaded at a rate of 8 N/min up to 12 N using custom built loading cups attached to a material testing machine (Instron 5800, High Wycombe, UK), which applied an axial load across the knee and ankle joints [14]. Two CCD cameras (100 mm lenses, GOM GmbH, Germany) mounted on a tripod were positioned horizontally in front of the loading fixture, at a distance of 42 cm, to provide a 15 x 12 mm field of view (7.5 x 10.9 μm resolution) with the depth of focus field of 1.2 mm. Along their axis, the two cameras were separated by 148 mm and they were rotated towards each other meeting at 25° angle on the bone surface of interest. Calibration was conducted by using a high-precision 15 mm x 12 mm panel (GOM GmbH, Germany). The sample was enlightened by two light-emitting diode lamps with polarised filters. During the loading, images of the medial side of the speckled tibiae surface were recorded at 1 N interval using the ARAMIS 5M System (GOM GmbH, Germany), with a resolution of 7.5x10.9 μm. Post processing of the images was done using 19x19 pixel square facets, with 15 pixels step facet. All the strains were computed with a computation size of 5 and a validity quote of 65%. Three images were taken in the un-deformed state in order to allow for the determination of the noise (inverse of accuracy) during the experiments. Max and mean strain on the medial surface were calculated.

### In situ Small Angle X-ray Scattering (SAXS) and Wide Angle X-ray Diffraction (WAXD) tensile tests

The ulnae of n = 5 TIMP3 Tg/Tg and n = 5 WT mice were dissected and soft tissue removed. All bones were maintained hydrated at 25°C with phosphate-buffered saline (PBS) after dissection and for the entire duration of the testing. A similar set-up that was previously used was applied for this test [38]. Bones were then inserted into a microtensile tester (Diamond Light Source (DLS)) that pulls the bones apart at both ends ensuring the sample remains at the same position with sub-micron precision. The bones were loaded in tension at a displacement rate of 2 μm∕s with a 110 N load cell (model 31- RDP Electronics, UK). The microtensile tester rig was positioned in beamline I22 at the DLS synchrotron radiation, such that SAXS/WAXD data were collected simultaneously with mechanical loading. During mechanical testing, samples were exposed to a microfocus X-ray (wavelength λ = 0.8857 Å, energy 14 KeV, size beam ~10μm^2^) for 0.2 s every 1% motor strain only during the elastic region of the load-displacement curve, limiting the total X-ray exposure of the bone. A high-speed Pilatus detector (Area Detector Systems Corporation) was positioned at approximately 1,000 and 250 mm from the sample, respectively, to collect SAXS and WAXD data.

The tissue strain was considered to be proportional to the motor strain. The analysis software Fit2D was used to convert the 2D data to 1D, by radially integrating over a 10° sector and a 20-pixel-wide sector, respectively oriented parallel to the direction of loading. The sample-to-detector distance and beam center were calibrated with standards. The fifth-order collagen peak and the (0002) apatite peak were found by fitting the 1D datasets with a Gaussian and a linear function. Changes in the position of the peak’s center divided by its location at zero, constituted the strain in the collagen fibrils and mineral.

### Murine bone cell culture

Bone cells were derived from cortical explants of mouse femur by adaptations of the outgrowth method [39]. The diaphyseal region of femurs of six 8wk-old TIMP3-Tg/Tg mice and four WT controls were stripped of all attendant periosteal soft tissue and were longitudinally bisected. The bone marrow was flushed out with Dulbecco's phosphate-buffered saline, without calcium and magnesium (PBS−) (Invitrogen., Paisley, U.K.) and each half was cut into fragments (approximately 1mm). After three washes in PBS− the bone fragments were transferred into tissue culture flasks; the 6 WT bone chips were places into 6 separate flasks and the 4 Tg/Tg bone chips were divided into 6 flasks (to ensure the resultant cultures were similar between eah genotype). Dulbecco's minimum essential medium (DMEM) containing 10% fetal bovine serum (FBS), 2 mM L-glutamine, and penicillin (100 IU/ml)/streptomycin (100 μg/ml) (Invitrogen.) was carefully added to each flask containing the bone chips and incubated at 37°C in a humidified 5% CO2 incubator. The cortical bone fragments were fed biweekly ensuring minimum disruption of outgrowing cells. After 30days of incubation, cells were washed in PBS− and released with 0.05% trypsin and 0.02% EDTA in special salt solution (Invitrogen, Paisley, UK). Only first passage cells (P1) were used for subsequent analysis.

### Cell counting

1000 cells/well were seeded onto a 48 well tissue culture plate and cultured for 18 days in DMEM. Monolayers were rinsed with ice-cold PBS twice and fixed in 4% paraformaldehyde for 15 minutes at room temperature. 500ul of PBS containing 50ul NucBlue™ Fixed Cell Stain (Invitrogen) was added to each well and incubated for 10 minutes at room temperature for dye incorporation. PBS containing dye was removed and replaced with 500μl fresh PBS. Twelve images of separate sections of each well were taken using Leica DMIRB inverted microscope under 450-490nm fluorescent emission settings. DAPI positive nuclei were counted in each image using Volocity™ software.

### Alkaline phosphatase activity

25,000 cells/well were seeded onto 12 well plates and cells were maintained in the presence and absence of osteogenic media (OM; containing 50ug/ml ascorbic acid and 2mM beta-glycerophosphate) with media changes every 3 days for 56 days. Cells were rinsed three times with PBS, digested with 0.1% triton X-100, scraped off the plate and the cell suspension sonicated (Ultrasonic disintegrator, MSE laboratories, Butte, MT). The protein assay was performed with the bicinchoninic acid (BCA) protein assay reagent (Pierce, Rockford, IL). Alkaline phosphatase activity was measured in the lysates as described previously [40]. Enzyme activity assay was performed in assay buffer (0.1 M diethanolamine, 1 mM MgCl2 and 2 mM p-nitrophenylphosphate pH 10.5) added to cell homogenates. A standard curve was prepared with p-nitrophenol solutions. Incubation of standards and samples was performed at 37°C for 10 min and the reaction stopped by adding 0.3 M NaOH. Reaction mixtures were transferred into 96-well plates and absorbance measured at 410 nm using a plate reader. Relative ALP activity is defined as nanomoles of p-nitrophenol phosphate hydrolyzed per minute per milligram of total protein. ALP activity was also determined by histochemical staining. Briefly, bone cells were fixed in 4% paraformaldehyde, rinsed and treated with substrate naphthol AS-TR which is subsequently hydrolysed in the presence of the enzyme. The introduction of fast blue dye produces a coloured precipitate.

### Total RNA extraction

Cells from the 6 bone chip flasks were pooled into 3 separate tubes (2 flasks pooled into 1) and 25,000 cells from these 3 tubes were seeded under basal or OM conditions in 12-well culture plates for 56 days (each plate consisted of n = 3 WT wells cultured with normal media, n = 3 Tg/Tg wells cultured with normal media, n = 3 WT wells under OM conditions and n = 3 Tg/Tg wells under OM conditions). This procedure was replicated three times independently to yield nine total RNA preparations for each treatment and genotype. Total RNA was isolated from cultured cells QIAZOL® reagent (QIagen, UK) according to the manufacturer’s instructions. Briefly, RNA was extracted at 56 days of culture. Cells were washed three times in ice cold PBS and lysed in QIAZOL reagent. The cell lysate was extracted with chloroform, and total RNA was precipitated with isopropanol. The pellet was washed in 70% ethanol, and resuspended in a small volume of diethyl pyrocarbonate (DEPC)-treated distilled water. Extracted RNA was treated with RNase-free DNase I (35 U/ml) for 30 min at 37°C and then re-precipitated using 3M sodium Acetate (Sigma Aldrich, UK). Total RNA concentration was measured by NanoDrop spectroscopy at 260nm and 280nm. A260/A280 ratio of 1.7–2.0 was considered to have sufficient purity. Samples were stored in a -80°C freezer until analysis.

### Gene expression

cDNA was prepared from the total RNA using the enzyme reverse transcriptase II. 100ng random hexamers (Qiagen) were added to 200ng total RNA, incubated for 10 minutes at 70°C and transferred to ice. A reaction mixture of 5x First Strand buffer, 0.1M Dithiothreitol (DTT), 10mM deoxynucleotide triphosphates (dNTPs) and 200 units Superscript II (all Invitrogen) was prepared in a total volume of 12μl and added to the RNA/random hexamer mixture. The reaction mixture was then run on a PCR cycler (Eppendorf) under the following conditions: 25°C for 10 minutes, 42°C for 50 minutes and 70°C for 15 minutes. The cDNA was then diluted 6 fold in DNase/RNase free H2O and stored at -20°C until use. Quantitative Reverse transcriptase polymerase chain reaction (qRT-PCR) was performed on cDNA to examine gene expression levels. PCR reactions were prepared in a total volume of 12μl, containing SYBR Green Jumpstart Taq readymix (20mM Tris-Hcl pH 8.3, 100mM KCl, 7mM MgCl2, 0.4mM dNTPs, Taq polymerase(Sigma Aldrich, UK) and DNAase, RNAase-free dH2O, 0.5μM forward primer, 0.5μM reverse primer and 0.6μg cDNA. The mixture was cycled on a PCR cycler (Biorad 384x) under the following conditions: 1 cycle of 94°C for 5 minutes, 40 cycles of 94°C for 10 seconds, 60°C for 15 seconds and 72°C for 30 seconds, followed by a final cycle of 70°C for 15 seconds. Results were analysed using the modified delta-delta CT method [41]. Specific primer sequences (sequence from 5’ to 3’, forward and reverse respectively) were: OCN (CATGAGGACCCTCTCTCTGC; TGGACATGAAGGCTTTGTCA), COL1A1 (TGGCCCCATTGGTAACGTTGGT; AGGACCTTGTTTGCCGGGTTCA), Runx-2 (TCTGCCGAGCTACGAAATGCCT; TGAAACTCTTGCCTCGTCCGCT), ALP (CCTTGAAAAATGCCCTGAAA; CTTGGAGAGAGCCACAAAGG). Melting temperatures for all of these sequences were 60°C.

### Statistical analyses

To compare WT with Tg/Tg samples Student’s t-tests were used. For in vitro data analysis, One Way ANOVA test was used. Statistical significance was calculated using GraphPad Prism software and p<0.05 was considered significant.

## Abbreviations

ADAMTS: A Disintegrin And Metalloproteinase with ThromboSpondin motifs
ALP: Alkaline phosphatase
ATF4: Activating Transcription Factor 4
BV/TV: Bone volume / Total volume
CA: Cortical area
Col2a1: Collagen type II alpha 1chain
Col1: Collagen type I
Cs.Th: Cross-sectional thickness
DIC: Digital image correlation
Ecc: Mean eccentricity
ECM: Extracellular matrix
GFP: Green fluorescent protein
IGF-1: Insulin growth factor-1
Ihh: Indian hedgehog
MA: Medullary area
Micro-CT: micro-computed tomography
MMI: Mean polar moment of inertia
MMP: matrix metalloproteinase
N: Newton
OM: Osteogenic medium
OCN: Osteocalcin
P14: 14 days-old postnatal
Runx-2: Runt-Related Transcription Factor 2
SAXS: Small angle X-ray scattering
Sox9: Sex-Determining Region Y-Box 9
TA: Total area
T.Pm: Total Perimeter
Tb.N: Trabecular number
Tb.Pf: Trabecular pattern factor
Tb.Sp: Trabecular separation
Tb.Th: Trabecular thickness
TIMP: Tissue inhibitor of matrix metalloproteinase
Tg/Tg: Homozygot transgenic
WAXD: Wide angle X-ray diffraction
WT: Wild-type
Wnt: Wingless-Type MMTV Integration Site Family
